# Relative Contribution of PIN-Containing Secretory Vesicles and Plasma Membrane PINs to the Directed Auxin Transport: Theoretical Estimation

**DOI:** 10.3390/ijms19113566

**Published:** 2018-11-12

**Authors:** Sander Hille, Maria Akhmanova, Matouš Glanc, Alexander Johnson, Jiří Friml

**Affiliations:** 1 Mathematical Institute, Faculty of Science, Leiden University, 2333 CA Leiden, The Netherlands; shille@math.leidenuniv.nl; 2 Institute of Science and Technology (IST) Austria, Am Campus 1, 3400 Klosterneuburg, Austria; axmahoba@gmail.com (M.A.); matous.glanc@gmail.com (M.G.); alexander.johnson@ist.ac.at (A.J.); 3 Department Experimental Plant Biology, Faculty of Science, Charles University, 12843 Prague, Czech Republic

**Keywords:** auxin, polar auxin transport, PIN transporters, secretion, 3D-SIM microscopy, mathematical modeling

## Abstract

The intercellular transport of auxin is driven by PIN-formed (PIN) auxin efflux carriers. PINs are localized at the plasma membrane (PM) and on constitutively recycling endomembrane vesicles. Therefore, PINs can mediate auxin transport either by direct translocation across the PM or by pumping auxin into secretory vesicles (SVs), leading to its secretory release upon fusion with the PM. Which of these two mechanisms dominates is a matter of debate. Here, we addressed the issue with a mathematical modeling approach. We demonstrate that the efficiency of secretory transport depends on SV size, half-life of PINs on the PM, pH, exocytosis frequency and PIN density. 3D structured illumination microscopy (SIM) was used to determine PIN density on the PM. Combining this data with published values of the other parameters, we show that the transport activity of PINs in SVs would have to be at least 1000× greater than on the PM in order to produce a comparable macroscopic auxin transport. If both transport mechanisms operated simultaneously and PINs were equally active on SVs and PM, the contribution of secretion to the total auxin flux would be negligible. In conclusion, while secretory vesicle-mediated transport of auxin is an intriguing and theoretically possible model, it is unlikely to be a major mechanism of auxin transport *in planta.*

## 1. Introduction

**Figure 1:**
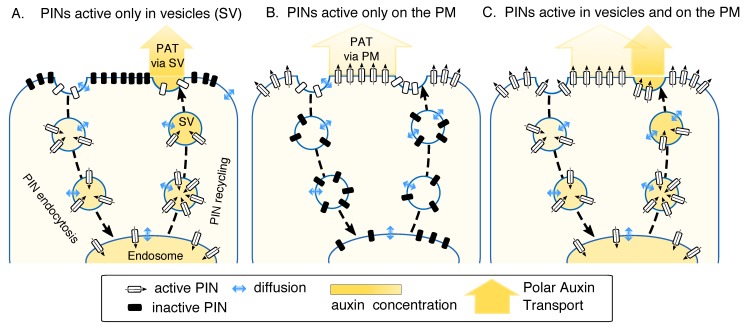
(**A**–**C**) Schematics of PIN-formed (PIN) endomembrane trafficking and possible auxin transport mechanisms.

The plant hormone auxin (indole-3-acetic acid; IAA) is subject to intercellular polar auxin transport (PAT) mediated by diffusion and the action of efflux and influx carriers. PAT generates local auxin maxima that are crucial for a plethora of developmental processes [1]. Therefore, studies into the mechanism of auxin transport and its regulation have had a prominent place in the auxin field. Auxin maxima-driven developmental events depend on the activity of the PIN-formed (PIN) IAA efflux carriers, which provide directionality to intercellular IAA transport through their asymmetric subcellular localization [1,2,3]. The PIN proteins constitutively cycle between the plasma membrane (PM) and endosomal compartments [4,5,6], and the developmental importance of this energetically demanding phenomenon has not been unequivocally explained. Three hypotheses have been suggested [7] to explain the requirement of PIN recycling: (1) recycling enables rapid relocation of PINs and thereby the rapid redirection of auxin transport; (2) PINs serve as IAA transceptors (transporters and receptors at the same time) and their recycling is important for the process of signal transduction; and (3) the PIN-containing secretory vesicles (SVs) are filled with auxin, which is released into the apoplast upon fusion of the SV with the PM. This process is analogous to neurotransmitter release in animals, and is important for the transport of auxin as suggested by previous reports [7,8] (Figure 1).

Several studies claimed to have proven the vesicular transport of IAA [9,10,11]. However, the validity of the evidence presented in most of these studies has been questioned by many researchers in the field, and thus the hypothesis of vesicular transport of auxin remains controversial [12,13]. A major reason this controversy exists is the inability to uncouple vesicular trafficking from PIN occurrence at the PM using existing cell biology methods. For example, if one could genetically or pharmacologically completely and specifically block the movement of PIN-containing secretory vesicles, it would be impossible to conclude whether the resulting reduction in auxin transport was caused by the lack of IAA secreting vesicles or by the lack of PINs at the PM.

Here, we have constructed a simple mathematical model to estimate the parameters under which auxin transport by intracellular vesicles could explain the measured net flux values of PAT. We show that a vesicular transport model would require at least 1000 times greater PIN activity than the conventional PM transport model to generate the same net flux values.

## 2. Results

### 2.1. Model Assumptions: PINs Can Be Active on the Endomembrane Vesicles Exclusively or in Addition to Their Activity on the Plasma Membrane (PM)

PAT is characterized by the sustained velocity of auxin over long distances (several millimeters, which is much greater than a typical cell length). The magnitude of this velocity is in the range of $1-10\frac{\mu m}{s}$ for different species and different types of plant tissue [14]. Arabidopsis root epidermal cells serve as a useful example because many of the physiological parameters that influence the PAT, and thus used in our calculations, have been experimentally measured in this cell type. Furthermore, vesicular auxin transport has been proposed to play a role in the transition zone of the root [9]. Thus, we chose to apply our model to this tissue. In the roots, auxin is transported towards the root tip inside the central cells (stele), whereas in the outer cell layer (lateral root cap cells and epidermal cells) it is transported in the reverse direction: from the root tip towards the shoot [15]. The velocity of this directed transport in *Arabidopsis* roots was found to be in a range $2-3\frac{\mu m}{s}$ [16]. We therefore assume that $v_{PAT}\geq 1\frac{\mu m}{s}$ in the epidermal cells in the root transition zone and estimated the permeability of PINs needed to yield this value.

**Figure 2:**
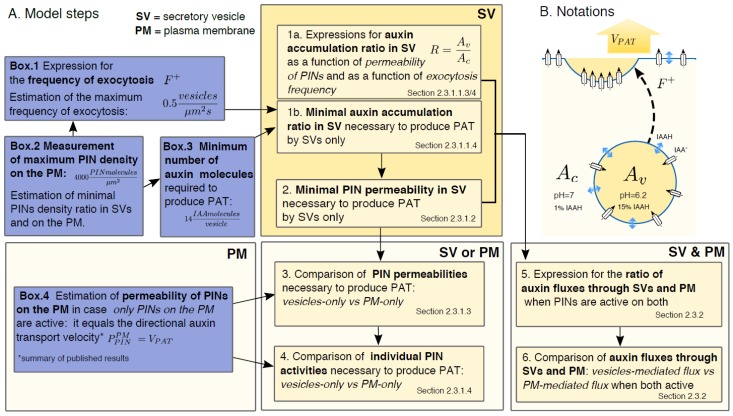
(**A**) Map of the model steps and usage of results from side-calculations presented in boxes; (**B**) scheme of the auxin transport into the secretory vesicle (SV) and illustration of variables used in the model.

In our model we consider only secretory/recycling vesicles (SVs) that fuse with the PM, and consider these secretory vesicles as the method of PIN delivery from the endosome to the PM. For such auxin-transporting vesicles we considered two hypothetical scenarios: (1) PINs transport auxin exclusively in these endomembrane vesicles, which we compare to the case when PINs are active only on the PM (published results) [17,18,19]; and (2) PINs transport auxin both in the vesicles and on the PM. The auxin permeability of a PIN-containing membrane (*P_PIN_*) remains undetermined. However, *P_PIN_* has been calculated previously for a scenario when PINs are active solely on the PM ($P_{PIN}^{PM}$) [17,18]. Therefore, the permeability of PINs in vesicles $P_{PIN}^{v}$ is a readout rather than input to our calculations. We calculated (1) the relative values of permeability $P_{PIN}^{v}$ and the individual activity of PINs proteins needed for the vesicular transport model to generate the same net flux as the PM model and (2) the relative contribution of the vesicular transport to the net flux, assuming that PINs activity is the same in the vesicles and in the PM. A plan of the model and performed calculations is presented in Figure 2A.

### 2.2. Short Summary of Model Results

(1) For the case when only vesicular PINs are actively transporting auxin, we derived equations for auxin concentration inside the vesicles, which depends on the permeability of the vesicle membrane to auxin due to PINs ($P_{PIN}^{v}\;\left[\frac{\mu m}{s}\right]$, the measure of how many auxin molecules per second are transported through $1{\;\mu m}^{2}$ area of a membrane when it is unit auxin concentration ($1\frac{\mathrm{mol}}{{\mu m}^{2}}$) outside the vesicle). At the same time, intravesicular auxin concentration determines the amount of auxin released into the cell wall. Therefore, we related macroscopic auxin transport velocity ($v_{PAT\;}\left[\frac{\mu m}{s}\right]$) to $P_{PIN}^{v}$ and calculated the coefficient of proportionality between these variables for the physiological values of the other parameters: $P_{PIN}^{v\text{-}only}\geq 42\cdot v_{PAT\;}$.

However, if PINs are only active on the PM, then PINs permeability $P_{PIN}^{\mathrm{PM}\text{-}only}≃v_{PAT\;}$ [17]. Thus, for the minimal velocity $v_{PAT}=1\frac{\mu m}{s}$, as observed in epidermal root cells [14], the lowest possible value of permeability is $P_{PIN}^{v\text{-}only}\;≃42\frac{\mu m}{s}$ for vesicular transport, which is much higher than the permeability required for PM transport $P_{PIN}^{PM\text{-}only}\;≃1\frac{\mu m}{s}$.

As the permeability is proportional to the density of PINs (the number of PIN molecules per unit area of the membrane), we generalized our equations via dividing permeability values by the density of PINs. The resulting quantity is the activity of individual PINs. We show that on the vesicle membrane PINs’ activity has to be 3 orders of magnitude higher than in the scenario where they are active only on the PM, in order to produce the same PAT velocity:

(1) $$\frac{\mathrm{PINs}\;\mathrm{activity}\;\mathrm{on}\;\mathrm{SV}\;\mathrm{only}}{\mathrm{PINs}\;\mathrm{activity}\;\mathrm{on}\;\mathrm{PM}\;\mathrm{only}}>\;4200.$$

(2) For the case when PINs are active both in vesicles and on the PM, our model provides the ratio of auxin amount passing through these two mechanisms depending on individual activities of PIN molecules in these domains:

(2) $$\frac{\mathrm{Auxin}\;\mathrm{flux}\;\mathrm{through}\;\mathrm{PM}}{\mathrm{Auxin}\;\mathrm{flux}\;\mathrm{through}\;\mathrm{SV}}=4200\;\cdot \frac{\mathrm{PINs}\;\mathrm{activity}\;\mathrm{on}\;\mathrm{PM}}{\mathrm{PINs}\;\mathrm{activity}\;\mathrm{on}\;\mathrm{SV}}.$$

Thus, if activities are equal, auxin flux through SVs contributes no more than 0.02% of the total flux.

**Conclusion:** The transport of auxin by vesicles is ~1000 times less efficient than through PINs active on the PM. Permeability values of PINs ($\;P_{PIN}^{v}$ and $P_{PIN}^{PM}$) still await direct measurements, but values as high as $P_{PIN}^{v}=42\frac{\mu m}{s}$, as estimated in our study, are unlikely. Therefore, this argues against vesicular transport of auxin in SVs as a major mechanism of directional auxin transport.

### 2.3. Detailed Model Description

#### 2.3.1. PINs Active Only in Vesicles. Comparison to the Case When PINs are Active Only on PM

List of parameters used in the model is provided in the Table 1. Assumptions taken throughout the model description are listed in Table 2.

##### 2.3.1.1. How Much Auxin Should a Vesicle with Active PIN Contain to Produce the PAT?

(1) Size of a Vesicle

The size of exocytotic vesicles that are considered to perform auxin transport is well known; they are spherical in shape and their diameter was reported to be d=0.06− 0.08 μm  [20] (see Table 1 for corresponding surface area and volume).

(2) Biochemical Constituents and Transport Processes, Equation for Auxin Concentration inside the Vesicle

The total concentration of auxin (IAA) is the mixture of anions IAA^−^ and protonated form (IAAH), and the ratio between them depends on pH of the solution:

$pH_{c}=7$ in cytoplasm, ~99% of IAA^−^, 1% of IAAH

$pH_{v}=5.5\;to\;6.5$ in vesicle, ~83–98% of IAA^−^, 17–2% of IAAH

$pK_{a}=4.8$ for IAA.

The fractions of IAA in anion form in cytosol and vesicle are denoted by $f_{ac}$ and $f_{av}$ respectively. The values were computed using:

(3) $$f_{av}=\frac{1}{1+10^{pK_{a}-pH_{v}}},$$

(4) $$f_{ac}=\frac{1}{1+10^{pK_{a}-pH_{c}}}.$$

Thus, $\left(1-f_{av}\right)$ and $\left(1-f_{ac}\right)$ are fractions of IAAH in vesicle and cytosol, respectively (see Figure 2B and Table 3 for IAA^−^ fractions depending on pH).

We denote:

$A_{c}$—cytoplasmic concentration of auxin [mol/L],

$A_{v}$—concentration of auxin inside the vesicle [mol/L].

Fast diffusion in the cytoplasm ($D≅670\;{\mu m}^{2}/s$ [17,25]) ensures fast mixing of IAA inside the small vesicle, that takes $\tau_{diff}\leq 10^{-6}$ s in vesicles of diameter d≤100 nm. Thus, IAA concentration inside the vesicle can be considered homogenous and increases proportionally to the flux through the membrane:

(5) $$\frac{dA_{v}}{dt}=\frac{1}{V_{v}}J_{in}^{net},$$

where $J_{in}^{net}$ is the net influx of IAA into vesicle (units [mol/s]), which has two components: transport of anions *IAA*^−^ by PINs and transport of *IAA*^−^ and *IAAH* by diffusion through the membrane:

(6) $$\;J_{in}^{net}=J_{PIN}^{IAA^{-}}+J_{diff}.$$

PINs transport anions IAA^−^ and are presumed to be permeable only in one direction (into vesicle) resulting in the unknown permeability $\;P_{PIN}^{v}$ of the vesicular membrane. We neglect dependence of PIN-mediated influx on intravesicular auxin concentration and assume that reverse permeability is zero. By doing so, we make PINs more efficient in our considerations than they can possibly be in reality. This approach is acceptable because our aim is to find the lower bound of the permeability of PINs which is able to produce the physiologically observed PAT transport. 

Thus, PIN-mediated transport is simplified to:

(7) $$\;J_{PIN}^{IAA^{-}}=P_{PIN}^{v}\;S_{v}\;f_{ac}\;A_{c}.$$

Diffusive flux across the membrane is governed by Fick’s law:

(8) $$\;J_{diff}=J_{diff}^{IAAH}+J_{diff}^{IAA^{-}}=P_{diff}^{IAAH}S_{v}\;\left(\left(1-\;f_{ac}\;\right)A_{c}-\left(1-\;f_{av}\;\right)A_{v}\right)+P_{diff}^{IAA^{-}}S_{v}\;\left(f_{ac}A_{c}-f_{av}\;A_{v}\right).$$

Diffusional permeability for IAAH is known: $P_{diff}^{IAAH}=\;0.5\;\mu m/s\;$ [18,24]. IAAH is assumed to have the same diffusional permeability $P_{diff}^{IAAH}$ in both directions.

Anion diffusion permeability is estimated to be much lower: $P_{diff}^{IAA^{-}}\leq \frac{P_{diff}\left(\mathrm{IAAH}\right)}{100}$ [24]. However, at $pH_{v}=7$, concentration of $[IAA^{-}]≅165\cdot [IAAH$] and thus diffusional flux of $IAA^{-}$. can be of the same order of magnitude as IAAH flux. Nevertheless, to simplify the equations we omit the term for $\;IAA^{-}$ diffusion. As diffusion of $IAA^{-}$ acts against PINs-mediated transport, this simplification also favors auxin accumulation into vesicles.

Combining Equations (7) and (8) and neglecting $J_{diff}^{IAA^{-}}\;$ result in the following expression for the net total flux:

(9) $$J_{in}^{net}=\;P_{PIN}^{v}\;S_{v}\;f_{ac}\;A_{c}+P_{diff}^{IAAH}S_{v}\;\left(\left(1-\;f_{ac}\;\right)A_{c}-\left(1-\;f_{av}\;\right)A_{v}\right).\;$$

Thus, Equation (5) can be rewritten as:

(10) $$\frac{dA_{v}}{dt}=A_{c}\cdot \alpha -\lambda \cdot A_{v},$$

where,

(11) $$\alpha =\frac{P_{PIN}^{v}\;S_{v}}{V_{v}}\;f_{ac}+\frac{P_{diff}^{IAAH}S_{v}}{V_{v}}\left(1-\;f_{ac}\;\right)\;,$$

(12) $$\lambda =\frac{P_{diff}^{IAAH}S_{v}}{V_{v}}\left(1-\;f_{av}\right).$$

(3) Maximum Loading of Vesicles with Auxin is Proportional to the Permeability of PINs

We assume that vesicles exist in the cytoplasm long enough that the internal vesicular auxin concentration reaches its maximum: steady state concentration $A_{v}$. The analytical solution of Equation (10) reads:

(13) $$A_{v}\left(t\right)=A_{c}\frac{\alpha }{\lambda }\left(1-e^{-\lambda t}\right)+e^{-\lambda t}A_{v}\left(0\right).$$

Thus, characteristic time to load vesicle from zero concentration till $A_{v}=A_{c}\cdot \frac{\alpha }{\lambda }\left(1-\frac{1}{e}\right)$ is $\tau_{load}=\frac{1}{\lambda }=4.6\;s$ for $pH_{v}=7$ (and faster for $pH_{v}<7$). For times $t\gg \tau_{load}$ vesicles will be fully filled. We consider fully filled vesicles because our aim is to find the *minimal* requirements for the vesicular transport, and partly filled vesicles would require *higher* permeability values to produce the PAT. 

At steady state, when $\frac{dA_{v}}{dt}=0$, Equation (10) simplifies to:

(14) $$A_{c}\cdot \alpha =\lambda \cdot A_{v}.$$

Thus, the number of IAA molecules (in moles) in one vesicle:

(15) $$N_{v}^{max}=A_{v}^{max}\cdot V_{v}=A_{c}\cdot V_{v}\frac{\alpha }{\lambda }.$$

Substituting α and λ by their expressions gives:

(16) $$N_{v}^{max}=A_{c}\cdot V_{v}\left[\frac{P_{PIN}^{v}}{P_{diff}^{IAAH}}\cdot \frac{f_{ac}}{1-f_{av}}+\frac{1-f_{ac}}{1-f_{av}}\right],$$

that shows that the number of auxin molecules loaded inside the vesicle is proportional to $P_{PIN}^{v}$.

We can also rewrite this equation in the form:

(17) $$N_{v}^{max}=A_{c}\cdot V_{v}\cdot R,$$

where,

(18) $$\;R=\frac{A_{v}^{max}}{A_{c}}=\frac{P_{PIN}^{v}}{P_{diff}^{IAAH}}\cdot \frac{f_{ac}}{1-f_{av}}+\frac{1-f_{ac}}{1-f_{av}}\;$$

is the accumulation ratio—the ratio of intravesicular IAA concentration to that in the cytoplasm surrounding the vesicle. Expressions for accumulation ratio and maximum loading of vesicles for different $pH_{v}$ are presented in Table 3.

(4) Lower Bound for Accumulation Ratio Necessary to Produce the PAT

In this section we estimated the auxin accumulation ratio R in vesicles, which is necessary to produce $v_{PAT}$ if auxin is transported via vesicles *only*. Auxin flux density (molecules passed per unit area per second) on the polar domain of the PM that corresponds to $v_{PAT}$ velocity is given by:

(19) $$\;\Phi_{cell\to apoplast}=v_{PAT}\cdot A_{c}\;\;\left[\frac{mol}{\;\mu m^{2}\cdot s}\right].$$

Maximum flux of auxin, that vesicles can carry through the PM is:

(20) $$\Phi_{v}=N_{v}^{max}\cdot F_{max}^{+}\;\;\left[\frac{mol}{\;\mu m^{2}\cdot s}\right],$$

where $F_{max}^{+}$ is the maximum exocytosis frequency (vesicles per second fusing with the unit area of cell face). This flux should be not lower than the yielded flux:

(21) $$\Phi_{v}\geq \Phi_{cell\to apoplast}.$$

This equation takes into account that diffusion diminishes the directional transport (see Box 4 for explanation).

Thus,

(22) $$\;N_{v}^{max}\cdot F_{max}^{+}\geq v_{PAT}\cdot A_{c}.$$

which gives, using Equation (17):

(23) $$\;R\cdot A_{c}\cdot V_{v}\cdot F_{max}^{+}\geq v_{PAT}\cdot A_{c}.$$

Consequently,

(24) $$R\geq \frac{v_{PAT}}{V_{v}\cdot F_{max}^{+}}.$$

Substituting $F_{max}^{+}$ with its expression (see Equation (1.7) in Box 1) $F_{max}^{+}=\frac{ln2}{S_{v}\;\cdot \;\tau_{1/2}}{\left(\frac{\rho_{PM}}{\rho_{v}}\right)}_{max}$, we can find the lower bound of the accumulation ratio, that is required for $v_{PAT\;}$:

(25) $$\;R\geq \frac{v_{PAT\;}\cdot S_{v}\;\;\cdot \;\;\tau_{1/2}}{V_{v}\cdot ln2}{\left(\frac{\rho_{v}}{\rho_{PM}}\right)}_{min}.$$

Note, that intracellular auxin concentration $A_{c}$ cancels (Equation (23)), which means that the equations are valid for any $A_{c}$. Nevertheless, we checked that vesicles will contain at least one molecule of auxin for the physiological value of $A_{c}$, a condition required for auxin transport to be theoretically possible (see Box 3). We also calculated the minimum accumulation ratio required for $v_{PAT\;}=1\;\mu m/s$ (Box 3).

Box 1 **Expression for $F^{+}$, frequency of secretory vesicles fusing with PM, and estimation of its maximum value $F_{max}^{+}$.**

In the following text we will consider only the polar domain of PM, which is the front membrane in the direction of PAT. It contains more PINs than the neighboring sides of the PM, and is visible by fluorescence microscopy [23]. The density of PINs depends on the rate of their delivery to PM by vesicles and on the rate of their removal. We assume that within the polar domain PINs are homogeneously distributed. Mass conservation for the number of PINs on a polar domain of the PM of area $S_{PM}$ then reads:

(1.1) $$\frac{dn}{dt}=n_{v}F^{+}S_{PM}-\beta \cdot n,$$

where n —number of PINs on PM, $n_{v}$—number of PINs in one vesicle, $F^{+}$ is frequency of exocytosis. PINs come to PM via $F^{+}S_{PM}$ vesicles per second. Removal of PINs is proportional to n with the decay coefficient β, which describes any possible decay mechanism (through endocytosis, diffusion to other PM domains, degradation etc.).

The decay coefficient β can be found from the following arguments. The equation for PINs that are already delivered to the PM (let us call them “old PINs”) and are only removed from the PM is the same as Equation (1.1) without exocytosis term:

(1.2) $$\frac{dn_{old}}{dt}=-\beta \cdot n_{old}.$$

The solution of this equation reads:

(1.3) $$n_{old}\left(t\right)=n_{old}\left(0\right)e^{-\beta \cdot t}.$$

Introducing half-life $\tau_{1/2}$, time when 50% of PINs has been removed ($n_{old}\left(\tau_{1/2}\right)=n_{old}\left(0\right)/2$), gives β:

(1.4) $$\beta =\frac{ln2}{\tau_{1/2}}.$$

$\tau_{1/2}=1.3\times 10^{4}\;s$, which has been measured by Jásik et al. ([23]) for functional PIN2-Dendra translational fusion protein in epidermal cells of Arabidopsis root. In their experiments, Jásik et al. ([23]) visualized “old PINs” separately from newly arriving PINs by use of photoconvertion of PIN2-Dendra.

Assuming that β is constant and does not depend on the method of PINs visualization, and assuming a steady state of PINs concentration on the PM, $\frac{dn}{dt}=0$, Equation (1.1) transforms to:

(1.5) $$n_{v}F^{+}S_{PM}=\beta \cdot n.$$

Equations (1.4) and (1.5) give us expression for $F^{+}$, the frequency of vesicle fusion with the PM:

(1.6) $$F^{+}=\frac{ln2}{S_{PM}\;\;\cdot \;\;\tau_{1/2}}\frac{n}{n_{v}},$$

We rewrite it using $\rho_{PM}=\frac{n}{S_{PM}}$, $\rho_{v}=\frac{n_{v}}{S_{v}}$—densities of PINs on the PM and vesicle membrane respectively:

(1.7) $$F^{+}=\frac{ln2}{S_{PM}\cdot \tau_{1/2}}\frac{n}{n_{v}}=\frac{ln2}{S_{PM}\;\cdot \;\tau_{1/2}}\frac{\rho_{PM}}{\rho_{v}}\frac{S_{PM}}{S_{v}}=\frac{ln2}{S_{v}\cdot \tau_{1/2}}\frac{\rho_{PM}}{\rho_{v}}.$$

Finally, introducing values for $S_{v}$ and $\tau_{1/2}\;$ gives:

(1.8) $$F^{+}=\frac{0.69}{1.1\;\times 10^{-2}{\;\mu m}^{2}\;\cdot 1.3\times 10^{4}{\;s}_{\;}}\frac{\rho_{PM}}{\rho_{v}}=\frac{\rho_{PM}}{\rho_{v}}\cdot 0.005\frac{1}{{\;\mu m}^{2}\;s}\;,\;$$

Introducing an estimation of minimum value for densities ratio ${\left(\frac{\rho_{v}}{\rho_{PM}}\right)}_{min}=0.01$ (see Box 2) into Equation (1.8) gives the upper bound for $F^{+}$:

(1.9) $$F_{max}^{+}=0.5\frac{vesicles}{{\;\mu m}^{2}\;s}.$$

The experimentally measured endocytosis rate gives an alternative estimate of the exocytosis rate.

In accordance with the above result, the measured rate of endocytosis is $\sim 0.5\frac{vesicles}{{\;\mu m}^{2}\;s}$ (Table 4). The total area of vesicles fusing with the PM in one cell has to be balanced by the area of endocytosed vesicles. Given that sizes of exocytotic and endocytotic vesicles are the same, the maximal rate of endocytosis gives a rough estimate of the maximal possible rate of exocytosis, which is in the same order of magnitude as our maximal theoretical estimate.

It is worth noting that the half-life of PINs $\tau_{1/2}$ was measured for “old” PINs and does not account for the possibility of “old” PINs being removed and brought back to the membrane via vesicles. This process would effectively reduce $\tau_{1/2}$ and allow for a higher $F_{max}^{+}$. However, based on our comparison with experimental values of endocytosis, we argue that $F_{max}^{+}$ cannot be much higher than $0.5\frac{vesicles}{\;\mu m^{2}\;s}$. This restriction comes from the physiological *in vivo* rate of endocytic vesicle formation (taking ~18–22 s per vesicle, see Table 4 [26]) and limited area of the cell membrane. Thus, as ${\left(\frac{\rho_{v}}{\rho_{PM}}\right)}_{max}=1$, $\tau_{1/2}$ can be reduced not more than by 100.

Box 2 **Estimation of minimal PINs density ratio on vesicles and the PM: ${\left(\frac{\rho_{v}}{\rho_{PM}}\right)}_{min}$.**

The lowest possible density of PINs on the vesicular membrane is one PIN molecule per vesicle: $\;{\left(\rho_{v}\right)}_{min}=\;\frac{1}{S_{v}}=50\frac{1}{{\mu m}^{2}}$.

The maximum PIN density can be calculated as follows: diameter of the globular 60 kDa protein is ~8 nm. If PINs on the PM are densely packed, their density is at most ${\left(\rho_{PM\;}\right)}_{max}≅\;2\times 10^{4}\frac{1}{{\mu m}^{2}}$, which is the maximum value of PIN density on the PM.

Thus,

(2.1) $${\left(\frac{\rho_{v}}{\rho_{PM}}\right)}_{min}=\frac{{\left(\rho_{v}\right)}_{min}}{{\left(\rho_{PM}\right)}_{max}}=0.002.$$

**Figure 3:**
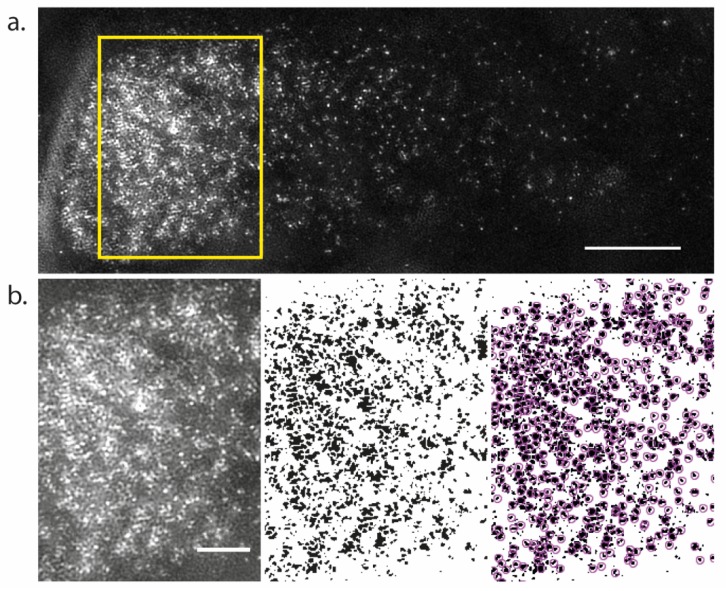
PIN2 translational fusion to Green Fluorescent Protein (PIN2-GFP) density measured by 3D structured illumination microscopy (SIM). (**a**) Example max projection of a 3D SIM image of the lateral membrane of root epidermal cells; (**b**) Left panel; the magnified yellow rectangle in (**a**). Middle panel; the image is made binary and subjected to watershed segmentation. Right panel; pink circles denote detected PIN2-GFP spots. Scale bars; (**a**), 5 µm, (**b**), 2 µm.

However, PINs probably cannot reach this maximum density *in vivo*, because numerous other proteins occupy space in the membrane. Also, quantification of PIN2-GFP in live epidermal cells of *Arabidopsis* root cells, observed using 3D structured illumination microscopy (SIM) with an x and y resolution of 106 nm and 104 nm respectively (Figure 3a), and an average acquisition speed faster than the average rate of endocytosis (see Methods & Table 4), showed that separated source-spots of GFP signal are always resolved with maximum density of $5.6\frac{\mathrm{spots}}{{\mu m}^{2}}$ (Figure 3b). Each of the spot contains at least one PIN-GFP protein. However, most spots are large: mean area of the spots is $0.034\pm 0.002{\;\mu m}^{2}$, max area $0.098{\;\mu m}^{2}$, min area $0.01{\;\mu m}^{2}$ (corresponds to resolution limit). Thus, most spots are likely to contain many PINs, which together fill at most 1/5 part of the membrane area (as $5.6\frac{1}{{\mu m}^{2}}\cdot 0.034{\;\mu m}^{2}=0.19$). This result argues that upper bound for the PIN density on PM is less than close-packing: ${\left(\rho_{PM\;}\right)}_{max}\leq 4\times 10^{3}\frac{1}{{\mu m}^{2}}$. Correspondingly,

(2.2) $${\left(\frac{\rho_{v}}{\rho_{PM}}\right)}_{min}=0.01$$

is a more realistic value of minimal PINs density ratio for epidermal root cells.

Box 3 **How many IAA molecules should be inside of one vesicle?**

Rewriting Equation (25) as inequality gives the accumulation ratio *R*, which is necessary for minimal PAT ($v_{PAT}=1\frac{\mu m}{s})$:

(3.1) $$R\geq \frac{v_{min\;}S_{v}\;\;\cdot \;\;\tau_{\frac{1}{2}}}{V_{v}\cdot ln2}{\left(\frac{\rho_{v}}{\rho_{PM}}\right)}_{min}=\frac{1\frac{\mu m}{s}\;2\times \;10^{-2}{\mu m}^{2}\;1.3\;\times \;10^{4}\;s}{2.7\;\times \;10^{-4}{\mu m}^{3}\;\cdot 0.69}0.002>2800.$$

This minimal required accumulation ratio is valid for any $A_{c}$. Nevertheless, we have to check, that vesicles will contain at least one molecule. In the volume of a vesicle (maximum 2.7 × 10^5^ nm^3^) there will be initially $N_{v}^{t=0}=A_{c}\cdot V_{v}=0.005$ molecules if cytoplasmic auxin concentration $A_{c}=30\;\mathrm{nM}$, which is a plausible estimate for the root epidermis [29]. For the lowest possible accumulation ratio R=2800, the number of molecules in one vesicle will be:

(3.2) $$N_{v}=A_{c}\cdot V_{v}\cdot R\approx 14,$$

which is a minimal requirement for vesicular transport.

##### 2.3.1.2. Minimal Permeability of PINs in Vesicles Necessary for Vesicular Auxin Transport

By combining Equations (18) and (25) we can find the relation between vesicular PINs’ permeability $P_{PIN}^{v\text{-}only}$ and the transport velocity $v_{PAT\;}$ in the case that PINs are only active in vesicles and thus all PAT is produced by vesicles:

(26) $$\;\frac{P_{PIN}^{v\text{-}only}}{P_{diff}^{IAAH}}\cdot \frac{f_{ac}}{1-f_{av}}+\frac{1-f_{ac}}{1-f_{av}}\geq \frac{v_{PAT\;}S_{v}\cdot \tau_{1/2}}{V_{v}\cdot ln2}{\left(\frac{\rho_{v}}{\rho_{PM}}\right)}_{min}.$$

Consequently,

(27) $$\;P_{PIN}^{v\text{-}only}\geq P_{diff}^{IAAH}\left(\frac{v_{PAT\;}\cdot S_{v}\cdot \tau_{1/2}}{V_{v}\cdot ln2}{\left(\frac{\rho_{v}}{\rho_{PM}}\right)}_{min}-\frac{1-f_{ac}}{1-f_{av}}\right)/\left(\frac{f_{ac}}{1-f_{av}}\right).$$

Thus, because $\frac{1-f_{ac}}{1-f_{av}}\leq 1$ can be neglected being much smaller than the first term $\frac{v_{PAT\;}\cdot S_{v}\;\cdot \;\tau_{1/2}}{V_{v}\cdot ln2}{\left(\frac{\rho_{v}}{\rho_{PM}}\right)}_{min}\geq 10^{4}$,

(28) $$P_{PIN}^{v\text{-}only}\geq P_{diff}^{IAAH}\cdot \frac{v_{PAT\;}\cdot S_{v}\cdot \tau_{1/2}}{V_{v}\cdot ln2}\frac{\left(1-f_{av}\right)}{f_{ac}}{\left(\frac{\rho_{v}}{\rho_{PM}}\right)}_{min}.$$

Grouping all parameters except $v_{PAT\;}$ and ${\left(\frac{\rho_{v}}{\rho_{PM}}\right)}_{min}$ into constant Κ gives:

(29) $$P_{PIN}^{v\text{-}only}\geq Κ\cdot v_{PAT\;}\cdot {\left(\frac{\rho_{v}}{\rho_{PM}}\right)}_{min}.\;$$

From Table 3 we find $min\left(\frac{1-f_{av}}{f_{ac}}\right)=0.006$ for $pH_{v}=7.0$, and using parameter values from Table 1, d = 0.08 μm, we calculate value:

(30) $$Κ={\left(\frac{P_{diff}^{IAAH}\cdot S_{v}\cdot \tau_{\frac{1}{2}}}{V_{v}\cdot ln2}\cdot \frac{1-f_{av}}{f_{ac}}\right)}_{min}=\frac{10^{2}\cdot 6\cdot 1.3\cdot 10^{4}}{8\cdot 0.69}\cdot \frac{6\cdot 10^{-3}}{2}≅4240\;.$$

Using $\frac{\rho_{v}}{\rho_{PM}}=0.01$ from Box 2, gives an estimate of the lower bound of PIN permeability in vesicles:

(31) $$P_{PIN}^{v\text{-}only}\geq 42\cdot v_{PAT\;}.$$

For $pH_{v}<7.0$ bounds for $\frac{P_{PIN}^{v\text{-}only}}{P_{diff}^{IAAH}}$ are provided in Table 3. For a pH value that we consider realistic, $pH_{v}=6.2$ permeability has to be higher: $P_{PIN}^{v\text{-}only}\geq 260\cdot v_{PAT\;}$.

##### 2.3.1.3. Comparison to the Case when PINs are Active Only on the PM: Permeability due to PINs has to be Much Greater on the Vesicles than on PM to Produce the Same Auxin Transport Velocity

It would be informative to compare our estimate of the permeability value (Equation (30)) $P_{PIN}^{v\text{-}only}\geq 42\cdot v_{PAT\;}$ with experimental measurements. Unfortunately, the permeability of PINs has never been measured directly in the intact tissues. Also measurements of the influx carrier permeability are not possible in explants like protoplasts, as PINs do not stay on the PM, but are instead internalized [18], resulting in no contribution to the efflux of auxin as in *in vivo* systems.

Fortunately, analogous estimates for PIN permeability have been done for the case when PINs are active only on the PM (see Box 4, [18]), which have shown that:

(32) $$\;P_{PIN}^{PM\text{-}only}≃v_{PAT}.$$

This relation was proved in theoretical studies using simple mathematical models and confirmed by computational models of multicellular tissues (see Box 4). It is determined under the assumption that PINs are active only on the PMs and facilitate auxin transport without any action from vesicles. Consequently, in epidermal cells of *Arabidopsis* root $P_{PIN2}^{PM}≃1\;\mu m/s\;$. 

We conclude from Equations (31) and (32) that if all other parameters are held in physiological range, to yield the same PAT velocity as PM PINs, vesicular PINs permeability has to be much higher: $\frac{P_{PIN}^{v\text{-}only}}{P_{PIN}^{PM\text{-}only}}\geq 42$. However, $P_{PIN}^{v\text{-}only}$ and $P_{PIN}^{PM\text{-}only}$ also depend on density of PINs on the membranes. To clarify this issue, in the next section we calculated the ratio of PINs activity, which is characteristic of individual transporters and which does not depend on the densities of PINs.

Box 4 **The permeability of PINs on the plasma membrane $P_{PIN}^{PM}$ equals the directional auxin transport velocity $v_{PAT}$.**

$P_{PIN}^{PM}$ can be derived from the following considerations, analogous to Equations (19)–(21). Auxin flux density due to PINs through the PM has the following expression because PINs are transporting anions IAA^−^ and depend only on its intracellular concentration $f_{ac}\cdot A_{C}$:

(4.1) $$\Phi_{PM}=P_{PIN}^{PM}\cdot f_{ac}\cdot A_{C},$$

that coincides with Equation (39).

It should be not lower than the PAT flux Equation (19): $\Phi_{PM}\geq \Phi_{cell\to apoplast}$, because other transport mechanisms, diffusion and non-polar transporters, are diminishing polar auxin transport velocity. The relationship between PAT velocity and the polar permeability accounting for the non-polar permeability was derived by Mitchison [17] (Equation (2)), which is discussed below in this box. Thus, an underestimate for $P_{PIN}^{PM}$ is obtained by equating the directional transport to transport by PM-PINs:

(4.2) $$P_{PIN}^{PM}\cdot f_{ac}\cdot A_{C}\geq v_{PAT}\cdot A_{c}.$$

Moreover, non-polar term is at least 100 less than $P_{PIN}^{PM}$. Consequently, we can consider

(4.3) $$P_{PIN}^{PM}\cdot f_{ac}=v_{PAT},$$

or, as $f_{ac}≅1,$ for pH = 7

(4.4) $$P_{PIN}^{PM}≅v_{PAT}.$$

Thus, for $v_{PAT}=1\;\mu m/s$, $P_{PIN}^{PM}≅1\;\mu m/s$.

In fact, References [17,19] have shown that for a file of cells transport speed is comparable to the efflux permeability, and this conclusion was confirmed by computer simulations of multilayered tissues [17,18,19]. In these classical works, it has been proven that the “advection” of auxin can be just a result of combined polar membrane transport and cytoplasmic diffusion. Thus, macroscopic advection velocity is limited either by polar membrane transport or by the rate of auxin’s transfer along the cell length; whichever value is lower. One can calculate that diffusion along the longest cell length (~ 100 μm) is faster than the measured velocity, proving that cytoplasmic transport does not limit the “macroscopic advection velocity $v_{PAT}$” [14]. In this case, polar membrane transport governs macroscopic velocity [17,19]. To show this, we provide derivation done by Mitchison [17].

**Derivation of** $P_{PIN}^{PM}$ **that would yield** $v_{PAT}=\;1\;\mu m/s$ **from theory developed by Mitchison [17]:**

Equation (2) from [17] reads:

(4.5) $$\frac{1}{v_{PAT}}=\frac{1}{p}+\left(1+\frac{2q}{p}\right)\frac{L}{2D,}$$

where $v_{PAT}$—macroscopic auxin velocity, p—polar efflux permeability, q—non-polar permeability (by diffusion), L—cell length, D—diffusion coefficient of auxin inside the cell. Because $\frac{L}{2D}\leq \frac{1\times \;10^{-4}m}{2\cdot 6.7\;\times \;\frac{10^{-10}m^{2}}{s}}=7.46\times 10^{4}\;s/m$, $v_{PAT}=1\times 10^{-6}\;m$/s:

(4.6) $$p=\frac{1+2q\frac{L}{2D}}{\frac{1}{\;v_{PAT}}-\frac{L}{2D}}\geq 1.1\cdot 10^{-6}m/s.$$

This equation shows that the efflux permeability $p≅{\;v}_{PAT}$, because $2q\frac{L}{2D}\ll 1$ for realistic maximum value of $q=10^{-6}\;m/s$. Note, that condition $\frac{1}{{\;v}_{PAT}}-\frac{L}{2D}\geq 0$ must hold, so that the value of permeability is positive, which is true for $v_{PAT}\leq \frac{2D}{L}=13.4\times 10^{-6}\;m/s$. The polar permeability value estimated in [17] is also in the order of $10^{-6}m/s$.

In principle, auxin-containing vesicles can also contribute to directional transport within the cell. However, as noted above, diffusion is already sufficient to transport auxin inside the cell, so such additional “acceleration” is not relevant for the macroscopic transport rate.

##### 2.3.1.4. Individual Activity of PINs Has to Be Much Higher on the Vesicles than on the PM to Produce the Same Auxin Transport Velocity

To compare the efficiency of transport, it is necessary to normalize permeabilities $P_{PIN}^{v\text{-}only}$ and $\;P_{PIN}^{PM\text{-}only}$ to the corresponding density of PINs. Membrane permeability due to PINs is proportional to the PINs’ density and can be expressed as a product of 1) density of transporters on the membrane $\rho_{PIN}\;[\frac{\mathrm{mol}}{{\mu m}^{2}}$] and (2) individual activity of one transporter protein $p_{PIN}[\frac{\mu m/s}{\mathrm{mol}/{\mu m}^{2}}$], which depends on affinity to auxin, electrical potential across the membrane ([19]), phosphorylation status, and any other parameters.

(33) $$\;P_{PIN}=\rho_{PIN}\cdot p_{PIN}.$$

The ratio of normalized permeabilities equals the ratio of the individual activity of PIN transporters situated in vesicles ($p_{v\text{-}only}$) and on the PM ($p_{PM\text{-}only}$): $\frac{p_{v\text{-}only}}{p_{PM\text{-}only}}=\frac{P_{PIN}^{v\text{-}only}}{\rho_{v}}\;\frac{\rho_{PM}}{P_{PIN}^{PM\text{-}only}}.$

Combining this equation and Equation (29) (equality form):

(34) $$\;P_{PIN}^{v\text{-}only}=Κ\cdot v_{PAT\;}\frac{\rho_{v}}{\rho_{PM}}\;$$

gives:

(35) $$\;\frac{p_{v\text{-}only}}{p_{PM\text{-}only}}=\frac{v_{PAT\;}\cdot Κ}{P_{PIN}^{PM\text{-}only}}\frac{\rho_{v}}{\rho_{PM}}\;\cdot \;\frac{\rho_{PM}}{\rho_{v}}.$$

Note that density ratio $\frac{\rho_{v}}{\rho_{PM}}$ cancels in this equation:

(36) $$\;\frac{p_{v\text{-}only}}{p_{PM\text{-}only}}=\frac{v_{PAT\;}\cdot Κ}{P_{PIN}^{PM\text{-}only}}.$$

By calculating the minimum of the right-hand side and using the fact that $v_{PAT\;}=P_{PIN}^{PM\text{-}only}\cdot f_{ac}$ (see Box 4), we can find the lower bound for the PINs activity ratio:

(37) $$\;\frac{p_{v\text{-}only}}{p_{PM\text{-}only}}\geq Κ\cdot f_{ac}\;$$

Introducing the minimal value of K (Equation (30)) and $f_{ac}=0.994$:

(38) $$\frac{p_{v\text{-}only}}{p_{PM\text{-}only}}>\;4200.$$

For $pH_{v}=6.2$, (see Table 3), the ratio is even higher: $\frac{p_{v\text{-}only}}{p_{PM\text{-}only}}>\;26600$. 

**Conclusion I**: For vesicle transport to be able to produce all of the observed IAA flux, the activity of PINs on the vesicle membrane has to be at least three orders of magnitude greater than that estimated for the case when PINs are only active on the PM. Our calculations show that transporting auxin directly through the PM is 1000 times more effective than by means of SVs. This, in our opinion, is an argument against SVs-mediated transport of auxin as a major mechanism of directional auxin transport.

#### 2.3.2. PINs Active Both in Vesicles and on the PM

Next, we derived the expression for the ratio of auxin fluxes through both mechanisms in the case that PINs are active on vesicles and the PM. Auxin efflux density caused by (active) PINs on the PM is:

(39) $$\Phi_{PM}=P_{PIN}^{PM}\cdot f_{ac}\cdot A_{C}.$$

Auxin efflux density caused by arriving PIN delivery vesicles (from Equation (20) using Equation (16) and omitting the term $\frac{1-f_{ac}}{1-f_{av}}$ as in Equation (28)):

(40) $$\;{\;\Phi }_{v}\;=N_{v}\cdot F^{+}≅\frac{P_{PIN}^{v}}{P_{diff}^{IAAH}}\cdot \frac{f_{ac}}{1-f_{av}}\cdot V_{v}\cdot F^{+}\cdot A_{c}.\;$$

Both effluxes are proportional to intracellular auxin concentration $A_{c}$, which is assumed to be the same at the PM and around SVs.

Dividing Equation (39) by Equation (40) gives the flux ratio:

(41) $$\;\frac{\Phi_{PM}}{{\;\Phi }_{v}}=\frac{P_{PIN}^{PM}\cdot f_{ac}A_{c}}{\frac{P_{PIN}^{v}}{P_{diff}^{IAAH}}\cdot \frac{f_{ac}}{1-f_{av}}\cdot V_{v}\cdot F^{+}\cdot A_{c}}=\;\frac{P_{PIN}^{PM}}{P_{PIN}^{v}}\cdot \frac{P_{diff}^{IAAH}\left(1-f_{av}\right)}{V_{v}}\cdot \frac{1}{F^{+}}.\;$$

Using Equation (33),

(42) $$\frac{\Phi_{PM}}{{\;\Phi }_{v}}=\frac{P_{diff}^{IAAH}\left(1-f_{av}\right)}{V_{v}}\cdot \frac{1}{F^{+}}\;\frac{\rho_{PM}}{\rho_{v}}\cdot \frac{p_{PM}}{p_{v}}.$$

Substituting $F^{+}$ by its expression from Equation (1.7) gives:

(43) $$\frac{\Phi_{PM}}{{\;\Phi }_{v}}=\;\frac{P_{diff}^{IAAH}\cdot \left(1-f_{av}\right)}{V_{v}}\frac{\rho_{v}}{\rho_{PM}}\frac{S_{v}\;\cdot \tau_{1/2}}{ln2}\frac{\rho_{PM}}{\rho_{v}}\frac{p_{PM}}{p_{v}},$$

where $\frac{\rho_{v}}{\rho_{PM}}$ cancel. Substituting with the constant *K* (Equation (30)) we reduce it further:

(44) $$\frac{\Phi_{PM}}{{\;\Phi }_{v}}=K\;\cdot f_{ac}\cdot \frac{p_{PM}}{p_{v}}.$$

The coefficient here is the same as in Equation (38):

(45) $$\frac{\Phi_{PM}}{\Phi_{v}}\geq 4200\;\cdot \frac{p_{PM}}{p_{v}},$$

where $\frac{p_{PM}}{p_{v}}$ is the ratio of individual PINs activity on the PM and vesicles if PINs are active on both membranes. Note that the densities of PINs cancel out and flux distributions between the two mechanisms depend only on the activity of PINs. Also Equation (38) can be derived directly from Equation (45) by assuming $\frac{\Phi_{PM}}{\;\Phi_{v}}=1$.

If the individual activity of PINs on the vesicle and on the PM are equal ($p_{v}=p_{PM}$), then,

(46) $$\frac{\Phi_{PM}}{\Phi_{v}}\geq 4200,$$

which means that the flux through the vesicle mechanism does not contribute more than 0.02% to the total flux.

For $pH_{v}=6.2$ the lower bound of the ratio is higher: $\frac{\Phi_{PM}}{{\;\Phi }_{v}}\geq 26600$, which implies that the contribution of the vesicular mechanism is less than 0.004%, which is negligibly small compared to PM flux.

The flux ratio $\frac{\Phi_{PM}}{{\;\Phi }_{v}}$ would be higher (favoring PM flux over SV-mediated flux) if:

- PINs half-life on the PM is higher;
- pH in the vesicle is lower;
- vesicles do not stay in the cytoplasm for long enough before fusing with the PM to be fully filled with IAA;
- the size of vesicles is smaller.

The opposite changes of parameter values would increase the SV-mediated contribution to the auxin flux (see Equation (43)).

**Conclusion II**: The activity of PIN transporters in the vesicle has to be at least 1000 times greater than on the PM to make a substantial contribution to the total directional auxin transport. When realistic physiological parameter values were used in the model, a factor greater than $10^{4}$ was determined.

## 3. Discussion

The hypothesis of vesicular secretion of auxin was postulated more than 15 years ago [7,8]. While several studies have claimed to provide experimental evidence to support this concept since [9,10,11], their conclusions are currently greatly debated in the field [12,13]. Hence the question whether vesicular secretion significantly contributes to intercellular auxin transport remains unresolved. Here, we took a modeling approach to estimate whether such mode of auxin transport is even theoretically possible.

First, we compared individual activities of PINs active exclusively on vesicles or on the PM that are necessary to yield the same PAT velocity (Equations (37) and (38)):

(47) $$\frac{p_{v\text{-}only}}{p_{PM\text{-}only}}\geq P_{diff}^{IAAH}{\left(\frac{6\cdot \tau_{1/2}}{d\cdot ln2}\cdot \frac{1-f_{av}}{f_{ac}}\right)}_{min}.$$

This ratio is valid for any PAT velocity and depends on the following measurable parameters: diameter of vesicles d, half-life time of PINs on the PM $\tau_{1/2}$, pH-dependent fraction of IAAH in a vesicle $\left(1-f_{av}\right)$, fraction of IAA^−^ in cytoplasm $f_{ac}$. Experimental parameter values (Table 1) gave us $\frac{p_{v\text{-}only}}{p_{PM\text{-}only}}>\;4200$, meaning that the individual activity of PINs on SVs ($p_{v}$) needs to be at least three orders of magnitude greater than the activity of PINs on the PM ($p_{PM}$) in order to produce the same auxin transport.

We also provide an estimate for the permeability of PINs in the vesicles required to yield $v_{PAT}=1\frac{\mu m}{s}$ in epidermal root cells: $P_{PIN}^{v}\geq 42\frac{\mu m}{s}$, which is not the measure of individual transporters, but characteristic of the unit area of the membrane. This value is much higher than any measured permeabilities to date [18,24]. The measurements of permeability due to PINs either on the PM, in vesicles or in both domains will allow one to draw precise conclusions from our model.

Calculation of permeability $P_{PIN}^{v}$ requires the knowledge of relative density of PINs proteins on vesicles and the PM. We provide measurement of PM density of PINs using SIM microscopy: $\rho_{PM}\leq 4\times 10^{3}\frac{1}{{\mu m}^{2}}$, which gives an estimate of minimal possible density ratio: ${\left(\frac{\rho_{v}}{\rho_{PM}}\right)}_{min}=0.01$ (Box 2).

Finally, we derived an expression that relates auxin flux driven by PINs on the polar domain of PM and auxin flux driven by PINs at the vesicles, if they are both active (Equation (43), using Equation (30)):

(48) $$\frac{\Phi_{PM}}{{\;\Phi }_{v}}=\frac{P_{diff}^{IAAH}\cdot \left(1-f_{av}\right)}{d}\cdot \frac{6\;\cdot \tau_{1/2}}{ln2}\;\cdot \frac{p_{PM}}{p_{v}}.$$

This shows that relative auxin fluxes through both mechanisms depend on: diameter of vesicles d, half-life time of PINs on the PM $\tau_{1/2}$, pH-dependent fraction of IAAH in a vesicle $\left(1-f_{av}\right)$ and individual activities of PIN proteins $p_{PM}$ and $p_{v}$.

The main parameter, namely PIN activity, has not been experimentally determined on the PM or in vesicles, which makes it impossible to find the actual ratio of fluxes *in vivo*. Nevertheless, by estimating the extreme values of the rest of parameters from available experimental data (Table 1), we were able to show that $\frac{\Phi_{PM}}{{\;\Phi }_{v}}>4200\;\cdot \frac{p_{PM}}{p_{v}}$. This expression suggests that if PINs were equally active both at the PM and on the SVs, the contribution of secreted IAA to the overall net IAA flux would be less than 0.02% at pH = 7 inside vesicles of maximal size, which is the maximal possible contribution in that case.

Moreover, instead of the half-life time of PINs on the PM $\tau_{1/2}$ and vesicle diameter, other measurable parameters can be put in this equation (using Equation (1.7), as done in Equation (42)): exocytosis frequency $F^{+}$, densities of PINs $\rho_{v,PM}$, pH-dependent fraction of IAAH in a vesicle $\left(1-f_{av}\right)$ and vesicular volume $V_{v}$:

(49) $$\frac{\Phi_{PM}}{{\;\Phi }_{v}}=\frac{P_{diff}^{IAAH}\left(1-f_{av}\right)}{V_{v}}\cdot \frac{1}{F^{+}}\frac{\rho_{PM}}{\rho_{v}}\frac{p_{PM}}{p_{v}}.$$

Interestingly, our theoretical estimate of maximum exocytosis rate Equation (1.7) coincides with experimentally measured maximum endocytosis rate: $F_{max}^{+}=0.5\frac{vesicles}{\;\mu m^{2}\;s}$ (see end of Box 1), which provides additional confirmation that the chosen parameter ranges are plausible. Equation (49) shows that the restrictions on frequency of exocytosis is one of the factors that limits the capacity of vesicles to release auxin at a high rate. Also limiting for SV-mediated transport is the fact that the density of PINs on the vesicle membrane cannot be higher than the density of PINs on the PM, which is almost at its possible maximum.

Our assumptions and calculations are based on the model of a PIN2-expressing root epidermal cell. We cannot rule out that vesicular transport of auxin could prevail in different cell types or physiological contexts. However, all parameters in Equations (48) and (49) except PINs activities cannot have zero values, thus, they would have to be changed by orders of magnitude to make vesicular auxin transport the dominant mechanism, causing massive changes in cell homeostasis. Therefore, we consider this unlikely and assume that our conclusions can be extrapolated to any cell type. If experimental values of parameters in any other cell type become available in the future, our equations can be used to resolve this question. 

If it was found that PINs transporters are active exclusively in vesicles and are inactive on the PM ($p_{PM}=0$), Equations (48) and (49) would become meaningless. Then the testable relation between the individual activity of PINs and the measurable parameters is (from Equation (28)):

(50) $$p_{v\text{-}only}=\frac{\;P_{PIN}^{v\text{-}only}}{\rho_{v}}=\frac{P_{diff}^{IAAH}}{\rho_{PM}}\cdot \frac{v_{PAT\;}\cdot 6\;\cdot \tau_{1/2}}{d\;\cdot ln2}\frac{\left(1-f_{av}\right)}{f_{ac}}.$$

However, the individual activity of PINs remains a technically demanding value to determine experimentally. Moreover, SV localization of a member of the AGCVIII kinase family, the action of which is required for PIN activity [30], has not been reported, arguing that PINs in the SVs are possibly inactive [13]. An additional argument that PINs are active at the PM is that increasing PINs incidence at the cell surface, e.g., by inhibiting their endocytosis leads to increased auxin efflux [31,32]. Overall, methods for separate measurement or perturbation of parameters in Equations (48) and (49) are needed to unambiguously distinguish between vesicular-mediated and PM-mediated auxin fluxes.

In conclusion, we have created a simple mathematical model to calculate the efficiency of PIN-mediated vesicular secretion of auxin compared to transport across the PM. Even under the most “vesicular transport-favoring” values of the parameters the vesicular transport of auxin was still determined to be several orders of magnitude less efficient compared to the membrane transport. Our calculations showed that PINs on the PM can produce auxin transport having much less individual activity than required for PINs in the secretory vesicles (SVs). Therefore, we consider it unlikely that PINs are active only in the secretory vesicles; and in case they are active on both PM and SVs, vesicular transport would play a negligible role in PAT.

## 4. Materials and Methods

### Measurement of PIN Density Using Structured Illumination Microscopy (SIM)

Live *Arabidopsis* roots samples were prepared as previously described by Johnson and Vert [26], where the imaging media was supplemented with 30% opti-prep and the coverslips were fixed on to the microscope slide. Cells in the elongation zone were imaged using an OMX BLAZE v4 3D SIM (Applied Precision). A 60 × 1.42 NA Oil Immersion objective and a 100 mw 488 nm laser was used. The microscope was aligned and calibrated using 100 nm tetra spark beads (Thermo Scientific) and found to have a working resolution of 106 nm and 104 nm in the x and y dimension and 336.9 nm in the z dimension. This SIM system was used due to its enhanced speed of acquisition, when compared to other systems, in order to minimize the effect of rapidly recycling proteins during acquisition. To overcome the curved and non-uniform shape of the roots, Z-stacks (with an optimal z spacing of 125 nm) were created to image the entire lateral membrane of the PIN2 polar domain. This is particularly important when imaging the lateral membrane next to the apical face of the cell, as it curves away from the coverslip. Depending on the shape of the cell, 1.625–3 µm stacks were made of each cell to ensure the entire lateral membrane region was captured, with an average acquisition time for the total z-stack of 17.7 s, with each z position having an average dwell time of 1.08 s. Each z-position image is based on 15 images generated from 3 different angles and 5 different SIM patterns using SOFTWORX software (Applied Precision). A maximum projection of the stacks was used for analysis. Images were made binary and subjected to watershed segmentation using Fiji [33]. PIN2-GFP spots were then detected using TrackMate [34]. The density of PIN2-GFP, mean, and maximum and minimum area of the PIN2-GFP spots were calculated from 12,343 spots in 9 different cells from 3 independent roots.

## Figures and Tables

**Table 1:** Values of parameters used in this study.

Parameter	Description	Value/Range	Ref.
d	Typical diameter of a vesicle	0.06–0.08 μm	[20]
$S_{v}=$ πd^2^	Surface area of a vesicle	$1.1–2.0\;\times 10^{-2}{\;\mu m}^{2}$	
$V_{v}=\left(1/6\right)$πd^3^	Volume of a vesicle	$1.1–2.7\;\times 10^{-4}{\;\mu m}^{3}$	
*pH_v_*	pH in the lumen of the trans-Golgi network/early endosome (TGN/EE)-derived vesicle	5.5–6.5	[21,22]
*pH_c_*	pH in the cytoplasm	7	[21,22]
$v_{PAT}$	Speed of directional plant hormone auxin (IAA) transport in epidermal cells of Arabidopsis root tip	2–3 μm/s	[14,16]
$\tau_{1/2}$	Half-life of PINs on the plasma membrane (PM)	$1.3\times 10^{4}\;s$	[23]
$\;{\left(\rho_{v}\right)}_{min}$	Minimum PIN density in a vesicle	$50\frac{1}{{\mu m}^{2}}$	One molecule per vesicle
$\;{\left(\rho_{PM\;}\right)}_{max}$	Maximum PIN density on a plasma membrane	$2\times 10^{4}\frac{1}{{\mu m}^{2}}\;\;$ $4\times 10^{3}\frac{1}{{\mu m}^{2}}$	Close-packing of equal spheres of 4 nm radius.Estimate based on experimental data, this study.
$P_{diff}\left(\mathrm{IAAH}\right)$	Diffusional permeability of IAA through membrane	0.5 μm/s	[18,24]

**Table 2:** List of assumptions *.

	Assumption	Effect on Efficiency of SVs to Transport Auxin	Effect on Efficiency of PM to Transport Auxin
1	Steady state/homogeneity: auxin fluxes and parameters that influence auxin fluxes are constant in time. Note, that experimentally measured values of parameters used in the model were also assumed to be constant when measured (Table 1): size of SV, pH in the cell and in SV, and listed below:		
1.1	PINs density on the PM equals an average density of PINs, being constant and homogenous along the whole polar side of the PM.		
1.2	PINs density on SVs is constant, all SV are identical and contain the same density of PINs.		
1.3	Intracellular auxin concentration near the PM is constant and homogenous.		
1.4	Intravesicular auxin concentration is homogenous.		
1.5	Removal of PINs from the PM is proportional to the PINs concentration on the PM and is a constant process in time, characterized by constant half-life of PINs on the PM. It doesn’t depend on the exocytosis rate.	Less efficient	No effect
2	SVs fuse with the PM and deliver all PIN proteins that they contain to the PM.	Less efficient	No effect
3	SV fills with auxin to its maximum concentration and then all auxin inside the SV is released outside the cell.	More efficient	No effect
4	Diffusion of anion form (IAA^−^) through the membrane (PM and SV) is negligible.	More efficient	-
5	PINs transport auxin (IAA^−^) only in one direction (inside the vesicle, outside the cell on the PM).	More efficient	More efficient
6	Non-polar auxin transport is neglected when equating auxin flux through PM or via SVs and directional auxin transport rate	More efficient, magnitude of correction is the same for PM and SVs	

* Validation of each assumption is provided in the text; most assumptions favor vesicular transport (increase auxin efflux mediated by SVs) and are taken to estimate minimal requirements for vesicular auxin transport.

**Table 3:** Dependence of the accumulation ratio **R** (Equation (18)) and maximum number of molecules in the vesicle $N_{v}^{max}$ (Equation (15)) on pH in the vesicle. The last column provides the lower bounds for $\frac{P_{PIN}^{v\text{-}only}}{P_{diff}^{IAAH}}$, required to yield directional transport of auxin $v_{PAT\;}$ depending on $pH_{v}$. $f_{Hv/c}$ —fraction of IAA^−^ in the vesicle/cytoplasm, $\left(1-f_{av/c}\right)$ —fraction of IAAH in the vesicle/cytoplasm.

$\;pH_{v}\;$	$\;f_{av}\;$	$\;1-f_{av}\;$	*R* *	$\;N_{v}^{max}\;$	$\;\frac{P_{PIN}^{v\text{-}only}}{P_{diff}^{IAAH}}\;$
5.5	0.833	0.167	$6.0\cdot \frac{P_{PIN}^{v}}{P_{diff}}+0.036$ **	$0.096\cdot \frac{P_{PIN}^{v}}{P_{diff}}$	$\geq 2330\frac{s}{\mu m}\;\cdot v_{PAT\;}$
6.2	0.962	0.038	$26.2\cdot \frac{P_{PIN}^{v}}{P_{diff}}+0.16$ **	$0.42\cdot \frac{P_{PIN}^{v}}{P_{diff}}$	$\geq 535\frac{s}{\mu m}\;\cdot v_{PAT\;}$
6.5	0.980	0.020	$49.7\cdot \frac{P_{PIN}^{v}}{P_{diff}}+0.3$ **	$0.80\cdot \frac{P_{PIN}^{v}}{P_{diff}}$	$\geq 280\frac{s}{\mu m}\;\cdot v_{PAT\;}$
7.0	0.994 =$f_{ac}$	0.006 $=1-f_{ac}$	$166\cdot \frac{P_{PIN}^{v}}{P_{diff}}+1$ **	$2.7\cdot \frac{P_{PIN}^{v}}{P_{diff}}$	$\geq 84\frac{s}{\mu m}\;\cdot v_{PAT\;}$

* For lower $pH_{v}$ accumulation ratios *R* have lower coefficient in front of $\frac{P_{PIN}^{v\text{-}only}}{P_{diff}^{IAAH}}$ because diffusion from the vesicle, that acts against PIN-mediated influx, is higher for lower $pH_{v}$. ** the right terms are always much smaller than the left terms and can be neglected, because $v_{PAT}\geq 1\frac{\mu m}{s}$ and thus $\frac{P_{PIN}^{v}}{P_{diff}}>84$.

**Table 4:** Calculation of rate of endocytosis (based on values from [27,28]).

**Endocytic Marker**	**DRP1C-GFP (Dynamin-Related Protein 1C-GFP)**	**CLC-GFP (Clathrin Light Chain-GFP)**
foci per µm^2^	3.54	3.48
standard deviation (SD)	0.62	0.55
average lifetime (s)	17.7	19.7
SD	8.8	6.8
foci per model cell (15 × 15 micron)	796.5	783
SD	139.5	123.75
**Endocytosis events per cell per second**	**DRP1C-GFP**	**CLC-GFP**
Average	45.0	39.7
max	105.2	70.3
min	24.8	24.9
**Maximum rate of endocytosis per second per µm^2^**	**0.467**	**0.312**

## Abbreviations

PM	Plasma membrane
SV	Secretory vesicle
IAA	Indole-3-acetic acid = auxin
PINs	PIN-formed transporters
TGN/EE	Trans-Golgi network/ early endosome
